# Integrating Elastic Tensor and PC-SAFT Modeling with Systems-Based Pharma 4.0 Simulation, to Predict Process Operations and Product Specifications of Ternary Nanocrystalline Suspensions

**DOI:** 10.3390/pharmaceutics13111771

**Published:** 2021-10-22

**Authors:** Andreas Ouranidis, Christina Davidopoulou, Kyriakos Kachrimanis

**Affiliations:** 1 Department of Pharmaceutical Technology, School of Pharmacy, Aristotle University of Thessaloniki, 54124 Thessaloniki, Greece; cdavidof@pharm.auth.gr (C.D.); kgk@pharm.auth.gr (K.K.); 2 Department of Chemical Engineering, Aristotle University of Thessaloniki, 54124 Thessaloniki, Greece

**Keywords:** nanosuspensions, elastic tensor analysis, process and material design space, PC-SAFT, spray drying, stabilizer selection, ball mill, interfacial Gibb’s energy, Pharma 4.0

## Abstract

Comminution of BCS II APIs below the 1 μm threshold followed by solidification of the obtained nanosuspensions improves their dissolution properties. The breakage process reveals new crystal faces, thus creating altered crystal habits of improved wettability, facilitated by the adsorption of stabilizing polymers. However, process-induced transformations remain unpredictable, mirroring the current limitations of our atomistic level of understanding. Moreover, conventional equations of estimating dissolution, such as Noyes–Whitney and Nernst–Brunner, are not suitable to quantify the solubility enhancement due to the nanoparticle formation; hence, neither the complex stabilizer contribution nor the adsorption influence on the interfacial tension occurring between the water and APIs is accounted for. For such ternary mixtures, no numeric method exists to correlate the mechanical properties with the interfacial energy, capable of informing the key process parameters and the thermodynamic stability assessment of nanosuspensions. In this work, an elastic tensor analysis was performed to quantify the API stability during process implementation. Moreover, a novel thermodynamic model, described by the stabilizer-coated nanoparticle Gibbs energy anisotropic minimization, was structured to predict the material’s system solubility quantified by the application of PC-SAFT modeling. Comprehensively merging elastic tensor and PC-SAFT analysis into the systems-based Pharma 4.0 algorithm provided a validated, multi-level, built-in method capable of predicting the critical material quality attributes and corresponding key process parameters.

## 1. Introduction

Comminution of poorly soluble active pharmaceutical ingredients (APIs) below the 1 μm threshold by wet media milling (WMM), followed by solidification of the obtained nanosuspensions via spray drying (SD), is an industrially feasible, scalable framework for solubility/dissolution enhancement [1,2]. Shear stress applied during WMM on crystals exhibiting surface defects [3] induces crack propagation, which results in fracture-creating new surfaces, increasing the Gibbs free energy [4]. Towards compensation of the interfacial tension abatement, the nanosuspensions swing to a thermodynamically unstable state, allowing for undesired Ostwald ripening and agglomeration events to occur [5]. The latter are invoked by electrostatic and/or steric stabilization attained by the participation of polymeric surfactants that improve the material’s surface wettability, bolstering the agglomeration activation barrier [6]. Moreover, thermodynamic material system stability and thus further processability is attained by atomizing the nanosuspensions against circulating heated gas streams, forcing evaporation of the solvent liquid phase [7]. The atomization process increases the available surface area, this time by facilitating the heat and mass transfer phenomena, allowing low processing operational temperatures in comparison to convective methods [8]. Although the solubility gain is generally evaluated by simply separating undissolved and dissolved components of the solidified formulated composition mixture, three main inconsistencies occur:

Firstly, for submicron ternary mixtures, the nanomechanical properties govern the physicochemical API behavior, i.e., the solubility and thermodynamic stability, which, in turn, define the macro-scale hierarchical functional attributes, such as bioavailability. Unwanted morphological transformations during product development processing and storage [9] also affect drug stability and nanosuspension handling, especially when shearing stress is applied. The piroxicam–succinic co-crystal formed via mechanical stress application, undergoing decomposition by shearing, represents such a case study [10]. Similar process-induced transformations remain both uncontrolled and unpredictable, thus mirroring the current limitations of our mechanochemical and atomistic level of understanding.

Secondly, nanoparticulate solubility determination becomes a complexed task of experimental practice when compared to the micronized scale. Elaborating, nanocrystals possess a high dissolution rate, and low-soluble nanoparticles cannot actually separate; consequently, the equilibrium of dissolution cannot be defined or validated and therefore the results obtained are poorly reproducible [11]. To counterbalance for the aforementioned ambiguity, the Noyes–Whitney and the Nernst–Brunner equations are frequently used for qualitative predictions of the saturation solubility increase tendency, bourn by the particle size comminution [12]. However, these conventional methods are also not suitable to accurately quantify the dissolution enhancement due to the nanoparticle formation, because neither the complex stabilizer contribution nor the adsorption influence on the interfacial tension occurring between the water and APIs is considered [12].

Thirdly the two aforementioned phenomena are interlinked, since the breakage process effecting the modification of the crystalline microstructure not only increases the particle surface, therefore enabling the supersaturation state, but also creates altered crystal habits of improved wettability, facilitated by adsorbed stabilizing polymers. These interfacial phenomena, dominant for solubility enhancement of ternary nanocomposites, typically consisting of API-polymeric stabilizer-water for dispersion medium, are therefore affected by crystal mechanical anisotropy, which depends on bulk crystalline properties and, in turn, affects the breakage-induced crystal habit changes. Currently, no numeric method exists to capture the correlation of the mechanical surface and bulk properties with the Gibbs interfacial energy of nanosuspensions, capable of informing the key process parameters associated with nanocomminution-related processes. Consequently, no simulation method has been developed to predict the product particle size distribution, which is the single most important critical quality attribute of the end product. Such a nanomechanically and physicochemically informed digital twin approach would facilitate the seamless technology 4.0 transfer to industrial settings, avoiding experimentation misfits and unnecessary expenditure of raw materials, energy, human and hardware resources, and more significantly contribute to the process-wide understanding and precision control of material systems.

Addressing the first challenge, elastic tensor analysis is a computational tool to quantify API stability during process implementation, utilized to study the interactions between APIs, excipients and co-formers, as well as the interactions between crystalline materials [13,14]. Regarding the second challenge, the drug nanoparticle core-shell was recently found to exist surrounded by a pseudo- and/or semi-solid phase structured by stabilizer and API placements, remaining in equilibrium with the solvent phase [15]. The validated existence of this iterated interface suggests a novel thermodynamic state, described by the stabilizer-coated nanoparticle’s dissolution Gibbs energy anisotropic minimization, offering a realistic prediction of the material’s system solubility, which can be quantified by the application of PC-SAFT model equations. Finally, merging elastic tensor and PC-SAFT analysis into a systems-based algorithm would provide a novel, multi-level, built-in algorithmic platform development capable of predicting critical WMM and SD process parameters, correlating the latter to key material properties, i.e., composition specifications (stabilizer selection) and quality attributes (particle size and moisture content). This paper refers to the experimentally validated global mechanistic study that bridges the aforementioned Pharma 4.0 enabling tools, to predict the processes parameters and their related product performance attributes, starting at the nanoscale level. Fenofibrate is utilized as a model drug, being a BCSII thermolabile API, well suited for solubility enhancement through WMD and SD processing [16].

## 2. Materials and Methods

### 2.1. Mechanical Properties Calculation by Elastic Constants Simulations

The crystal lattice energy of Fenofibrate was minimized utilizing the GULP code [17] while the mechanical properties, such as the shear (G) and the bulk (K) moduli, the Hill averages projected in the three dimensions for the Young (E) moduli and compressibility, were calculated by the second derivative matrices. In order to simulate the elastic properties of the API, the ElATools algorithm [14] was utilized to perform facile analysis of the second-order crystal stiffness elastic tensor leveraging on the transformation law. The latter is a recently deposited open script compiled by Fortran, able to resolve the calculation of the basic mechanical properties such as the bulk, Young and shear modulus, universal and Chung–Buessem anisotropy index, Cauchy pressure, logEuclidean anisotropy parameter and Poisson’s and Pugh’s ratio, exploiting the averaging schemes of Reuss, Voigt and Hill. In order to validate the results, the spatial dependence of Young’s modulus and the compressibility were additionally calculated by the ELATE tool for the analysis of the elastic tensors [18], which is available online at http://progs.coudert.name/elate (accessed online 9 September 2021). Moreover, the Vickers hardness and fracture toughness were calculated according to Mazhnik et al. (2019) [19] using the USPEX Hardness tool (available online at https://uspex-team.org/online_utilities/hardness3/, accessed on 9 September 2021). The Bond Work Index was calculated by the empirical Equation (1) given by the tentative method developed by Gent et al. (2012) [20]:

*BWI*_0_*=* 50 *−* 6*VH*^2^ − 0.003*VH +* 9.6937 (1)

where *BWI* is the bond work index and *VH* the Vickers hardness in kg/mm^2^.

### 2.2. PC-SAFT Model Implementation

Perturbed-Chain Statistical Associating Fluid Theory was used to predict the thermodynamic properties and phase equilibria by statistical mechanical methods [21]. Via PC-SAFT, the residual Helmholtz energy was calculated as the sum of the contributors in Equation (2) [21,22]:

(2) $$a^{res}=a^{hc}+a^{dis}+a^{assoc}$$

where *a^hc^*, *a^dis^* and *a^assoc^* are the contributions of the hard chain repulsive interactions, the van der Waals interactions and the associating interactions, respectively. PC-SAFT approximates the molecules of a component *i* as the chains constitute spherical segments. The calculation of the *a^hc^* and *a^dis^* contributors are required as the input parameters for each pure component *i*, the number of segments per chain *m_i_*_,_ the segment diameter *σ_i_* and the dispersion energy *ε_i_ k^−^*^1^ where *k* is the Boltzmann constant. For the API–polymer–water nanosuspension mixture considered in this work, the cross-interaction parameters were determined using the Berthelot–Lorentz combining rules (Equations (3) and (4)):

(3) $$\sigma_{ij}=\frac{\sigma_{i}+\sigma_{j}}{2}$$

(4) $$\varepsilon_{ij}=\sqrt{\varepsilon_{i}\varepsilon_{j}}\;\left(1-k_{ij}\right)$$

Τhe dimensionless values of the binary interaction parameters *k_ij_* were adjusted to fit the experimental solubility data [23,24]. For the calculation of the association contribution, the association energy *ε^AiBi^ k^−^*^1^ and the dimensionless association volume *κ^AiBi^* were considered. The association Helmholtz energy contribution *a^assoc^* refers herein to the formation of hydrogen bonds in the aqueous environment of the mixture. In Table 1 and Table 2, the iterated pure component and binary interaction parameters are indicated, respectively.

The association Helmholtz energy *a^assoc^* was calculated by Equation (5) [28,29].

(5) $$a^{assoc}=\underset{i}{\sum }x_{i}\left[\underset{A_{i}}{\sum }\left(lnX^{A_{i}}-\frac{X^{A_{i}}}{2}\right)+\frac{1}{2}M_{i}\right]$$

where *i* is the mixture component, *x_i_* is the mole fraction of the mixture, *M_i_* is the number of association sites in the molecule of the component *i* and *X_Ai_* is the mole fraction of the *i*th component’s molecules not bonded at the association site *A*. *X_Ai_* can be calculated using Equation (6).

(6) $$X_{Ai}={\left[1+N_{AV\;}\underset{j}{\sum }\underset{B_{j}}{\sum }\rho_{j}X^{B_{j}}\Delta^{A_{i}B_{j}}\right]}^{-1}$$

where *j* represents the second component whose molecules participate in the *i*–*j* association pair, *B_j_* is all the association sites of a *j*th component’s molecule, *X^Bj^* is the *j*th component’s molecular fraction not bonded at site *B*, *ρ_j_* is the molar density and Δ*^AiBi^* is the association strength (Equation (7)):

(7) $$\Delta^{A_{i}B_{j}}=d_{ij}^{3}g_{ij}\left(d_{ij}\right)\kappa^{A_{i}B_{j}}\left[\exp\left(\frac{\varepsilon^{A_{i}B_{j}}}{kT}\right)-1\right]$$

The term *d_ij_* represents the average temperature-dependent diameter of the *i*th and *j*th component’s molecules, *T* is the temperature and *g_ij_*(*d_ij_*) is the radial pair distribution function expressed for mixtures of a hard-sphere reference system [21]. For our API–polymer–water ternary mixture, the self-association hydrogen bonding Helmholtz energy contribution of the water was considered significant, due to the low value of the HPMC molar fraction (*x_HPMC_ <* 0.01) (Equation (6)), the poor association bonds between the water and the Fenofibrate and the non-existent self-association.

### 2.3. Process Model

The model was implemented in Aspen Plus V9.0 (Aspen Technology, Burlington, MA, USA). The software was utilized to simulate the interdependent parameters of the investigated pharmaceutical processes, namely, WMM and SD, by converging the energy and the material system mass balances. Both the wet mill and the spray dryer calculations were performed in the steady state. For the simulation mathematical model’s solution, the Sequential Modelling (SM) method was chosen, which is also the default strategy in the Aspen Plus software. Via the SM approach, each block’s output variables were calculated in sequence using the specific input parameters and zero degrees of freedom.

#### 2.3.1. Predicting the Ball Mill Key Process Parameters and the Output Quality Material Attributes

Mechanistic and empirical knowledge contribute to the forming of unchangeable factors, i.e., the length to diameter ratio, the values of which are produced empirically and are dimensionless. The ball mill design space prediction requires the consideration of adjustable parameters as well as empirical factors and assumptions taken from the associated bibliography. The device’s length-to-diameter ratio value was considered in connection to the chamber’s rotational speed, being a dependent parameter effecting changes to the final product specifications. For changeable parameters such as the type, size of the grinding balls and rotational speed, these also affect the physical properties specifications. A novel algorithm was created, to estimate the dependent variables for each capacity. The required input decision process parameters for the ball mill design space prediction, except the mass of the API’s media to be grinded, are the mill’s diameter and the mass grinding media specific power. To model the grinding process, Rittinger’s law of comminution was employed (Equation (8)), being appropriate for the nanoscale:

(8) $$E=C_{R}\;\left(\frac{1}{d_{P}}-\frac{1}{d_{f}}\right)$$

where *E* is the specific power required for the milling operation, *C_R_* is the Rittinger’s coefficient, and *d_f_* and *d_p_* the characteristic particle size before and after the crushing process. For *d_p_* and *d_f_*, the most used is the *d*_80_ value. As Equation (8) implies, the energy required for a reduction in the micro solid particle size appears proportional to the surface increase. Rittinger’s coefficient is calculated according to Equation (9):

(9) $$C_{R}=0.5\;C_{B}\sqrt{d_{BI}}$$

where *C_B_* is Bond’s coefficient calculated in Equation (10), and *d_BL_* is the limit of the Bond range.

(10) $$C_{B}=10BWI$$

#### 2.3.2. Distribution Functions

The Weibull distribution of the RRSB (Rosin–Rammler–Sperling–Bennet) distribution edition was considered, presenting the cumulative fraction of the particles being lesser than or equal against a given diameter (Equation (11)):

(11) $$Q\left(d\right)=1\;–\;exp\left\{-{\left(\frac{d}{d_{63}}\right)}^{n}\right\}$$

where *n* is the parameter of dispersion determined by the steepness of distribution *x* from Equation (12):

(12) $$n=\frac{ln\left(\frac{ln\;0.75}{ln0.25}\right)}{lnx}$$

and *d*_63_ was estimated based on the specified median value from Equation (13).

(13) $$d_{63}=\frac{d_{50}}{{\left(ln2\right)}^{1/n}}$$

#### 2.3.3. Process SD Model

**Figure 1:**
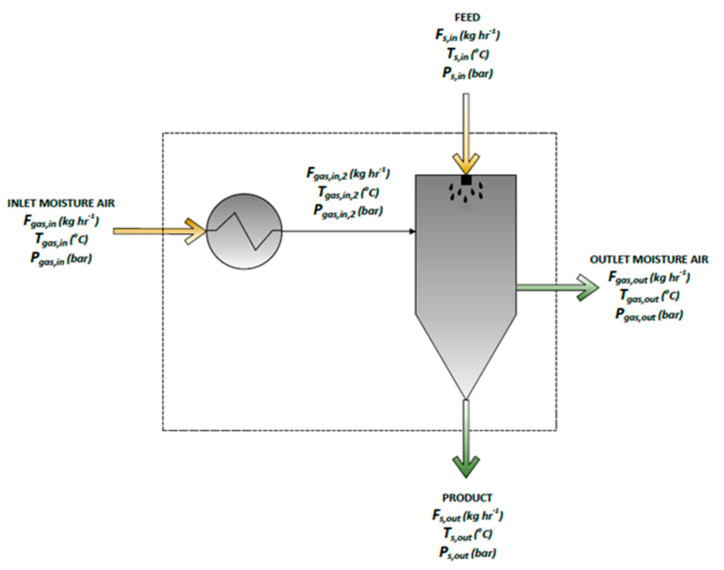
Process parameters of the spray dryer in the Aspen Plus simulation.

The SD’s design space prediction followed the previous WMM’s approximation strategy. Specifically, the critical process parameters considered in this work for the SD model are the drying gas flow (*F_gas_*_,*in*_), temperature (*T_gas_*_,*in*_), pressure (*P_gas_*_,*in*_) and the nozzle orifice diameter of the device (*D_n_*) (Figure 1). The drying gas flow rate *F_gas_*_,*in*_ is pre heated at the duty *Q_in_*. The API nanosuspension enters the spray tower with a *F_s_*_,*in*_ flowrate, while the gas and solid stream leave the dryer at an *F_gas_*_,*out*_ and *F_s_*_,*out*_ flow rate, respectively. The general mass balance can be shown by Equation (14):

*F_gas,in_* + *F_s,in_* = *F_gas,out_* + *F_s,out_* (14)

The general mass balance can be written otherwise as shown in Equation (15):

(15) $$Y_{in}\;F_{gas,dry}+X_{in}F_{s,dry}=Y_{out}F_{gas,dry}\;\;X_{out}F_{s,dry}$$

where *Y_in_* and *X_in_* are the dry basis moisture of the inlet drying gas and API nanosuspension, *Y_out_* and *X_out_* are the dry basis moisture of the outlet streams, respectively, and *F_gas_*_,*dry*_ and *F_s_*_,*dry*_ are the drying gas and API pure solid stream flow rates, which are considered constant. The overall enthalpy-energy balance is given in Equation (16):

0 = *H_gas,in_* + *H_s,in_* + *Q_in_* − *H_gas,out_* − *H_s,out_* − *Q_loss_* (16)

The enthalpy of the drying gas is calculated using the dry gas flow and moisture, the heat capacity of the dry gas, *C_p_*_,*gas*,*dry*_, and of the moisture *C_p_*_,*w*_ and the latent heat of vaporization at the reference state, Δ*H_v_*_,0_, as shown in Equations (17) and (18).

*H_gas,in_* = *F_gas,dry_* ((*C_p,gas,dry_* + *Y_in_C_p,w_*) *T_gas,in_* + *Y_in_ΔH_v,0_*) (17)

*H_gas,out_* = *F_gas,dry_* ((*C_p,gas,dry_* + *Y_out_C_p,w_*) *T_gas,out_ + Y_out_ΔH_v,0_*) (18)

The enthalpy calculation of the nanosuspension using the solid loading and the heat capacity can be calculated according to Equations (19) and (20).

*H_s,in_* = *F_s, dry_* (*C_p,s,dry_* + *X_in_C_p,w_*) *T_gas,in_* (19)

*H_s,out_* = *F_s, dry_* (*C_p,s,dry_* + *X_out_C_p,w_*) *T_gas,out_* (20)

#### 2.3.4. SD Evaporation Model

The rate of evaporation, which is also referred to as the drying rate of a saturated droplet, is calculated according to Equation (21).

(21) $$\dot{v=\frac{M}{M_{0}}=2\;F\;\frac{X_{w}}{X_{w,crit}}}-\left(2\;F-1\right){\left(\frac{X_{w}}{X_{w,crit}}\right)}^{2}$$

where *M* and *M*_0_ are the normalized and the initial evaporation rate of a saturated droplet, *ν* is the normalization factor, *F* is a shape factor ranging from zero to unity, *X_w_*_,*crit*_ is the critical moisture content and *X_w_* the residual moisture content, both on a wet basis.

#### 2.3.5. SD Particle Formation Model

The nanoparticle diameter was considered separately for the two phases of drying. The second phase begins when the critical moisture content is reached, and at that moment the droplet is considered more as a moisture solid particle. For moisture content, *X_w_ > X_w_*_,*crit*_, the particle diameter is calculated as in Equation (22).

(22) dp=ms 1ρs+Xdρl6π13

where *X_d_* is the dry basis moisture content, *ρ*_s_ is the solid API density, *ρ_l_* is the liquid density and *m_s_* is the solid API particle mass within the shrinking droplet, which is considered constant assuming non-existent interactions between the droplets. It is also assumed that the product’s diameter remains constant after the time point when the critical moisture content is reached.

### 2.4. Phase Equilibria Model

The API’s solubility in the aqueous solvent (*x_i_*, mol mol^−1^) was described by thermodynamic SLE (Solid-Liquid-Equilibria) terms via Equation (23) [30]:

(23) $$x_{i}=\frac{1}{\gamma_{i}}exp\left\{-\frac{\Delta h_{0i}^{SL}}{RT}\left(1-\frac{T}{T_{0i}^{SL}}\right)-\frac{\Delta C_{p,0i}^{SL}}{R}\left[ln\left(\frac{T_{0i}^{SL}}{T}\right)-\frac{T_{0i}^{SL}}{T}+1\right]\right\}$$

where $T_{0i}^{SL}$, $\Delta h_{0i}^{SL}$ and $\Delta C_{p,0i}^{SL}$ are the melting temperature, the melting enthalpy, and the difference between the solid and liquid heat capacity of the pure API, respectively. Herein, *T* represents the temperature, *R* the ideal gas constant and *γ_i_* the activity coefficient, calculated according to Equation (24):

(24) $$\gamma_{i}=\frac{\varphi_{i}}{\varphi_{i}^{0}}$$

where $\varphi_{i}$ and $\varphi_{i}^{0}$ are the fugacity coefficients of the API in the mixture and in the pure API form, respectively. The fugacity coefficients are derived from the calculated PC-SAFT-Helmholtz energy *a^res^*, using the relevant thermodynamic relationships.

### 2.5. Gibbs Energy Enhancement and Solubility Model Implementation

In the solid API nanosuspension, a decrease in particle size, increase in temperature and/or addition of the stabilizer favor the dissolution process [15,31,32]. The solubility enhancement achieved is proposed to be predicted by an interfacial Gibbs energy thermodynamic model [15]. In a dissolution reaction, the molar Gibbs energy will be described via Equation (25):

(25) ΔG=−RTlnK

where *R* the ideal gas constant, *T* the temperature and *K* the dissolution equilibrium constant, as from Equation (26):

(26) $$K=\prod_{n}{\left[A_{n}\right]}^{k_{n}}$$

The term *[A_n_]* indicates the equilibrium concentration of component *A_n_* and *k_n_* is its reaction coefficient. The API’s particle size decrease and the interfacial semi-solid phase presence in the nanocrystals’ surface created via the stabilizer’s addition lead to a decrease in the dissolution’s Gibbs free energy, and thus to solubility enhancement [15,33]. This Gibbs energy enhancement is described herein as the sum of two energy contributors, namely, the Gibbs energy of the API’s nanoparticle surface $\left(G_{m}^{s}\right)$ and the corresponding one of the interfaces caused by the stabilizers $\left(G_{m}^{i}\right)$ (Equations (27)–(30)):

(27) $$GEE=\Delta G_{1}-\Delta G_{2}=RTln\frac{K_{2}}{K_{1}}$$

(28) $$GEE=G_{m}^{s}+G_{m}^{i}$$

(29) $$G_{m}^{s}=\frac{2\gamma V_{m}}{r}\left(1-\frac{C}{r}\right)$$

(30) $$G_{m}^{i}=1.7\frac{\varepsilon_{API}\sigma_{API}}{\rho_{stab}\Delta \left(\sigma_{stab-API}\right)\left(\varepsilon_{stab-API}\right)m_{stab}}\frac{\gamma V_{m}}{r}$$

where *K*_2_ is the dissolution equilibrium of the API nanoparticles, *K*_1_ is the corresponding one of the initial (large) nanoparticles, *γ* is the surface tension calculated via Equation (31) [34], *V_m_* is the molar volume, *r* is the particle’s characteristic size, *C* is a parameter calculated via Equation (32), *ρ_stab_* is the stabilizer’s molecular density calculated via Equation (33), Δ is the distances between the molecular layers taken from bibliography [35] and *m_stab_* is the number of segments per chain of the stabilizer according to the PC SAFT theory. The molecular parameters *ε_API_* and *σ_API_* are the PC-SAFT parameters (Table 1) and *ε_stab-API_* and *σ_stab-API_* are calculated via Equations (3) and (4).

(31) $$\gamma =-\frac{0.33kT}{{\left(\frac{V_{m}}{N_{A}}\right)}^{2/3}}\left[\ln\left(\frac{S_{0}}{55.6}\right)+5\right]$$

(32) $$C=1.5{\left(\frac{V_{m}}{N_{A}}\right)}^{1/3}$$

(33) $$\rho_{stab}=\frac{\rho_{b,stab}}{M}N_{A}$$

where *S*_0_ is the solubility of Fenofibrate in pure water, *ρ_b_*_,*stab*_ is the stabilizer’s bulk density and *M* is the stabilizer’s molecular weight.

## 3. Results

### 3.1. Mechanical Properties

**Figure 2:**
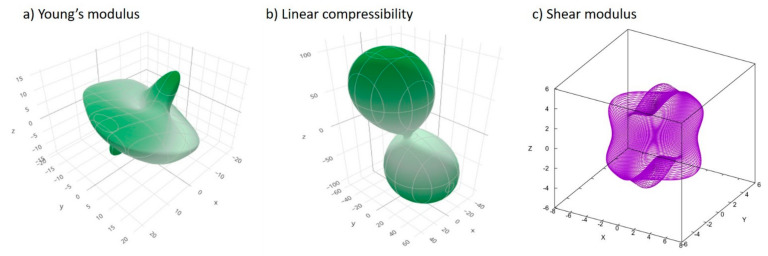
Nanomechanical properties in spatial-dependence functions: (**a**) Young’s modulus; (**b**) linear compressibility index; (**c**) shear modulus of the Fenofibrate nanocrystals.

For crystalline materials exhibiting anisotropy, elastic properties are of pivotal importance for the introduction of the material system to the process simulator engine. First principles simulations have therefore been performed by Elate, ElaTools and USPEX to describe the mechanoelastic profile of Fenofibrate, herein summarized by Table 3, while the related Figure 2 shows the spatial distribution coordinates of the Young modulus, linear compressibility index and shear modulus index tensors. In detail, a high universal anisotropy index is observed, exhibiting the orientation dependence of Young’s modulus, predicating the presence of bulk and crystal surface imperfections [36]. Young’s modulus and linear compressibility are single unit vectors, by convention here α functions, being parametrized by dual angles in the spherical coordinates 0 ≤ *θ* ≤ π, 0 ≤ *φ* ≤ 2π (Equation (34)):

(34) $$\alpha =\left\{\left.\begin{array}{c} sin\left(\theta \right)cos\left(\varphi \right)\\ sin\left(\theta \right)sin\left(\varphi \right)\\ cos\left(\theta \right) \end{array}\right\}\right.$$

Young’s modulus quantification, herein being the first-order derivative of tensile stress to axial strain, consequently measures the API’s material elasticity in tension, i.e., the opposite directional stiffness [14], exhibiting actual anisotropic behavior and high fracability. The ELATE Figure 2a,b and ElaTools Figure 2c visualization tools project the latter into the Euclidean space, as the parametric surfaces, respectively. Linear compressibility (LC) [22], Poisson’s ratio (PR) for auxetic material and the anisotropic elastic modulus were assessed as they constitute critical elastic properties, when associated with negative algebraic values owing to stress or strain [14]. The latter was implemented by validating the non-negative linear compressibility and non-negative (auxetic) Poisson’s ratio (NPR) [37], in order to discard the bizarre possibility of monodirectional material expansion.

**Figure 3:**
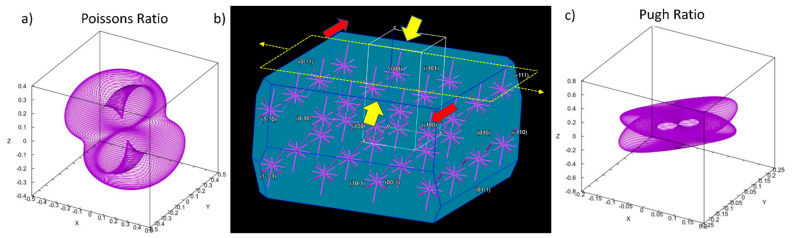
Comprehensive schematic analysis of lattice displacement: (**a**) spatially dependent Poisson’s ratio; (**b**) crystal lattice morphology. Heavy yellow arrows represent the tensile stress acting towards the (100) Miller direction, while light yellow arrows represent the mass expansion with regard to the perpendicular axial direction to the (001) Miller plane, upon simulating mass displacement in order to calculate the Poisson’s ratio. Red arrows indicate the favored displacement directions oriented on the (001) Miller indexed plane; (**c**) spatially dependent Pugh ratio.

The Fenofibrate crystal bulk when loaded by tension is expected to extend into the applied force direction, accompanied by the relevant lateral deformation. The inherited displacement was quantified by Poisson’s ratio, which, in turn, is physically rendered as the negative ratio index under uniaxial stress of the transverse to the longitudinal strain. The aforementioned attributes of the lattice arrangement are represented by a visual analysis of the elastic tensors in Figure 3, becoming comprehensive and exploitable towards our milling material input parametrization.

In addition, according to our calculations, Fenofibrate exhibits a positive polycrystalline Poisson’s ratio of 0.36 GPa towards the (001) Miller index plane. Elaborating the former calculation, when tensile stress acts internally to the aforementioned plane direction, namely, towards the (100) Miller direction (see heavy yellow arrows in (Figure 3b), then the crystal lattice of the API tends to expand with regard to the perpendicular axial direction of the (001) Miller plane (see light yellow arrows in the same Figure), by elsewhere shrinking its bulk to conserve its mass, thus pertaining more to a ductile rather than brittle fracture regime [14]. In order to corbel the aforementioned finding, the Pugh ratio, being the dimensionless fraction of the shear (G) to bulk (B) modulus [38], was calculated and found to be 0.49, thus exhibiting a marginal prevalence of the latter. Therefore, Gauchy pressure was finally utilized to aid the ductile-to-brittle transition quantification assessment according to the Pettifor methodology [39], proposing the existence of angular covalent bonds, Figure 3b, that pertain to the ductile profile.

### 3.2. WMM Model Expansion and Experimental Validation

The *BWI* predicts the energy requirements of comminution assisting the estimation of energy losses, critical for the Rittinger-based estimations. The *BWI* is calculated by correcting the experimentally projected *BWI*_0_ with correlating factors, namely, *F*_1_ being the open circuit milling factor, *F*_2_ the dry milling factor, *F*_3_ the mill diameter factor, *F*_4_ the inlet particle size factor and *F*_5_ the inlet size reduction ratio; thus, the corrected *BWI* can be calculated via Equation (35):

(35) $$ΒWI=BWI_{0}\left(\;\Pi_{i=1}^{5}F_{i}\right)$$

**Figure 4:**
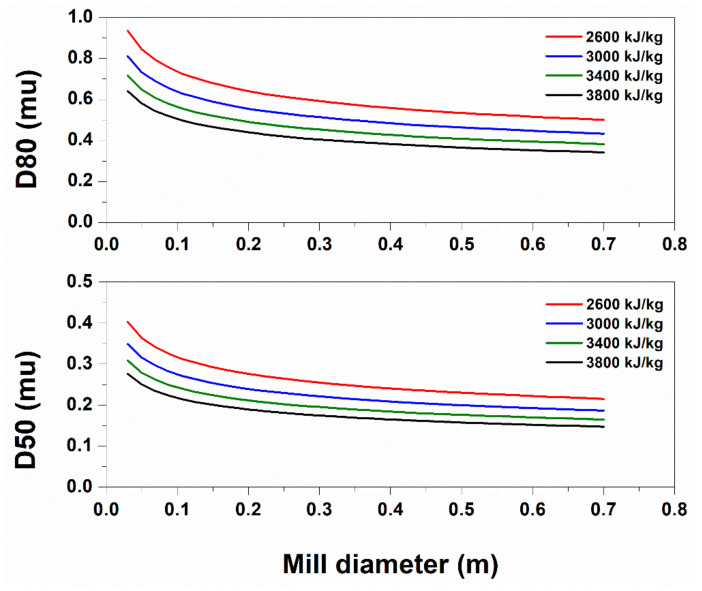
Change in the D80 nanoparticulate diameter (nm) with regard to the milling jar diameter design specification in m.

Figure 4 is based—independent from the mass—on Rittinger’s Law, the Rosin–Rammler equation and the initial *D*80, and therefore appears not to be linked to capacity.

**Figure 5:**
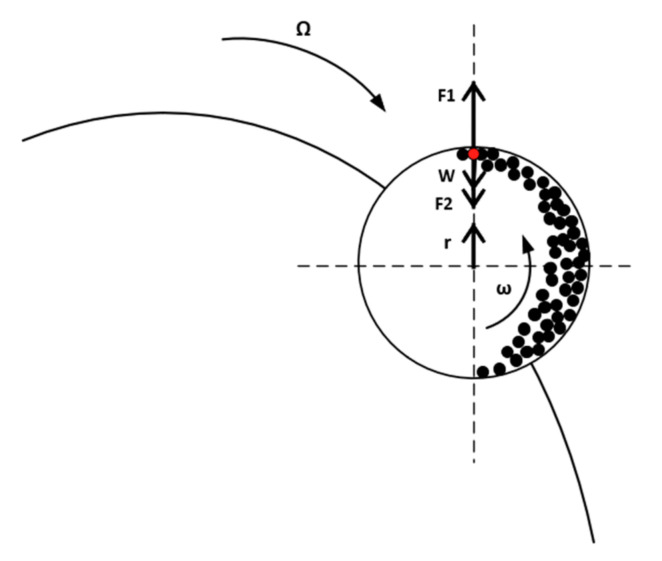
Trajectory schematic of the planetary ball mill, disc and jar, exhibiting their respective rotational speed and the relative forces applied to the grinding media.

Beginning with the characteristic product size, there exists a predictable design specification area of the mill’s advantageous operation. By increasing the diameter after a specific value, a non-additional specific power input is needed to achieve the same result, and by decreasing the diameter of the mill after a specific threshold, no requirement of a specific power increase encumbers the same response. The operational area of the ball mill was validated between the specific values from our previous grinding Fenofibrate experimental results, delivering submicron crystals of characteristic (*D*50) size between 300 and 400 nm [16]. Given this and according to Figure 4, for the Aspen engine to simulate this level of grinding by using a *D_m_ =* 10 cm mill diameter, an input-specific energy of *E =* 2600 kJ/kg or *E =* 702 kWh/ton is evidently required. In Figure 5, a trajectory approximation of the planetary ball mill principle of function is depicted.

The jar chamber executes the circular trajectory with the power of the device being correlated to the mass throughput *m_B_* by relationship (36), while the first and the angular chamber speed being connected by the torque $\vec{\tau }$, as per Equation (37) [40]:

(36) $$P=Em_{B}$$

(37) $$P=\left|\vec{\tau }\right|\omega$$

The torque is the product of distance from the center of the mass $\vec{r}$ and the force $\vec{\Sigma F}$ is applied to the latter (Equation (38)):

(38) $$\vec{\tau }=\vec{\Sigma F\;}\vec{r}\;\to \;\left|\vec{\tau }\right|=\left|\vec{\Sigma F}\right|\left|\vec{r}\right|cos\varphi$$

*φ* being the angle between the distance and the force vector. Generally, the net force applied in each unit of mass *m_i_* inside the mill in each coordinate is shown by Equation (39):

(39) $$\vec{\Sigma F}=\vec{F_{2}}+\vec{W}+\vec{F_{1}}\;$$

where $\vec{F_{2\;}}$ is the centripetal force, $\vec{W}$ the weight of the mass in the red spot (Equation (41)) and $\vec{F_{1}}$ the centrifugal force (Equation (40))

(40) $$\vec{\left|F_{1}\right|}=m_{i}\omega^{2}r$$

(41) $$\vec{\left|W\right|}=m_{i}g_{\;}$$

According to the Pulverissete-7 Fritch, Germany, planetary ball mill technical data (https://www.fritsch-international.com/sample-preparation/milling/planetary-mills/details/product/pulverisette-7-premium-line/technical-details/, accessed on 9 September 2021), a 95-G centripetal force is acting upon the material inside the chamber (Equation (42)):

(42) $$\vec{\left|F_{2}\right|}=95gm_{i}$$

Assuming that the internal mill mass is distributed towards the cylinder’s internal surface in such a way that the total mass is approximately a thousand and a half times the mass located in the red spot (Equation (43)):

(43) $$m_{B}=\frac{1500m_{i}}{t_{B}},$$

where *t_p_* is the mass flow residence time inside the mill, considered equal to 1 h—the mill’s batch experimental cycle time [16]. More specifically, in the highest spot inside the mill (the red spot), the net force can be calculated from Equation (44):

(44) $$\vec{{\left|\Sigma F\right|}_{c}}=\vec{{\left|F_{2}\right|}_{c}}+\vec{{\left|W\right|}_{c}}-\vec{{\left|F_{1}\right|}_{c}}$$

The critical angular speed of the chamber *ω_c_* turns the net force $\vec{{\left|\Sigma F\right|}_{c}}$ equal to zero. To conduct proper grinding, the chamber’s angular speed must fulfill the condition in Equation (45) [41]:

(45) $$\omega =0.65\omega_{c}$$

The revolution speed of the chambers will be equal to the speed, and its vector has the opposite direction [42] (Equation (46)):

(46) $$\vec{\Omega }=-\vec{\omega }$$

Conclusively, combining Equations (36)–(46) to the red spot (see Figure 5), for the actual rotation speed *ω*, the total mass inside the mill is found. Table 4 shows the obtained results from the Aspen Plus simulation in comparison with the experimental results.

### 3.3. SD Modeling Expansion and Experimental Validation

**Figure 6:**
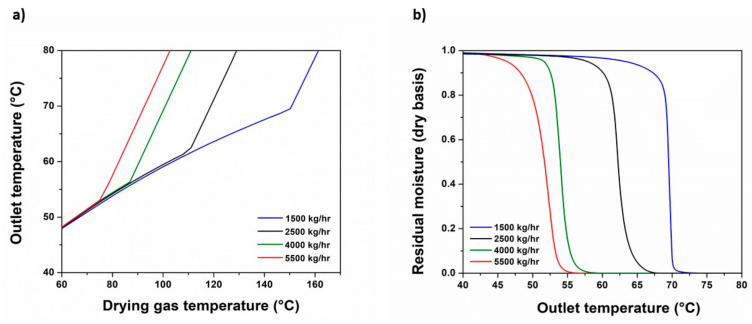
SD model calculated process parameters: (**a**) equilibrium temperature and inlet air temperature relationship for several inlet air flows; (**b**) residual moisture of the nanoparticles relative to the equilibrium temperature.

The SD process is considered as a form of quenching attributed to the evaporative cooling of the solute due to solvent evaporation. In the current work, SD is used to condense the nanosuspension by controlling the drying temperature in order for the solid stream not to exceed the Fenofibrate’s low melting point of 80 °C [23]. Several sensitivity analyses were conducted for the Fenofibrate/HPMC/water nanosuspension to determine the suitable design space that fulfills the latter statement and simultaneously fits the experimental data [16]. The critical process parameters considered in the analysis are the temperature, the pressure and the mass flow of the drying gas, i.e., the moisturized air and the nozzle orifice SD diameter (Table 5). Figure 6 shows the moisturized air temperature and the residual moisture within the outlet (equilibrium) streams’ temperature relationship, for several air flow rates during the SD process.

For each air flow rate selected when exceeding a specific threshold, the equilibrium temperature becomes increasingly sensitive to the inlet air temperature changes. In Figure 6b, the residual moisture of the particles in the end of the SD process vanishes at an equilibrium temperature equal to that of the critical temperature exhibited by Figure 6a, for the given air flow rate. Considering this phenomenon, the temperature behavior explains the SD progress; hence, reaching a specific moisture content during drying, i.e., the critical moisture content, the drying droplet transits to a moisturized particle. Upon the droplet reaching this critical moisture content, the drying rate is falling until reaching equilibrium with the drying gas, and the moist particle’s surface temperature becomes more prone to the temperature rise, owing to more intensified heat transfer phenomena [43]. This point is dependent not only from the temperature but also from the air mass flow rate. The relative humidity of air with a specific moisture content is falling as the temperature rises, resulting in a higher water capacity. For inlet air temperatures below the critical point, the moisture content of the nanoparticles in the end of the SD process is not wholly evaporated due to the air’s poor moisture capacity. Thus, the rising air mass flow rate diminishes the temperature requirements to achieve total moisture evaporation. The sensitivity analysis matches the key parameters, in line with those of our experimental work, and are presented in Table 5.

**Figure 7:**
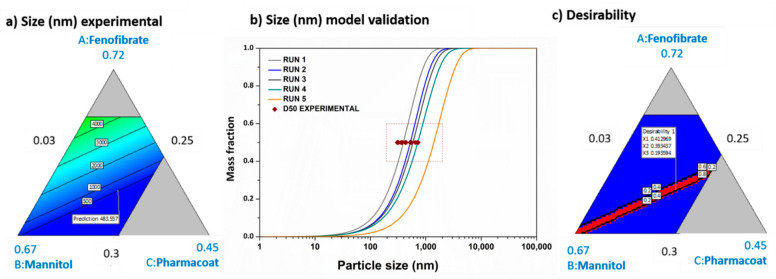
Experimental validation of the obtained end-product’s critical quality attribute regarding size and desirability index: (**a**) ternary contour diagram of the correlations among the formulation mixture variables, Fenofibrate, Pharmacoat 603 and Mannitol, with overlaid diagrams demonstrating the effect on size; (**b**) results of the particle size distribution obtained by the simulation engine and experimental fitting of D50, presented by the red squares; (**c**) experimental design space optimization based on the desirability levels.

The triadic contour diagrams of the ternary component mixture experimental correlations among the contribution levels of the formulation variables, Fenofibrate, Pharmacoat 603 and Mannitol, against fixed levels of temperature, showing the effects on size for the final SD particles, are described in Figure 7a,b, which show the results by in silico prediction and the fitting of the experimental values, while Figure 7c exhibits the overlaid diagrams of the desirability level, resolving the design space studied.

The numeric element denoted by each apex describes the maximum level of the referred variable, whereas the three lines (AB, BC and CA) joining the vertex points represent the combination of A, B and C; i.e., they represent the two components or binary mixtures at a fixed level of the other variable, which is shown in the center on the line.

According to the factorial sensitivity analysis, increasing the drying gas mass flow rate and the temperature or reducing the nozzle orifice diameter corresponding to the droplet size or the drying gas pressure, result in the formation of finer powder particle sizes (see Table 5 and Figure 7b). The particle morphology is mainly dependent on the drying rate; i.e., increasing the drying rate enhances the heat and mass transfer phenomena occurring in the droplets and the particles, producing hollow and/or smaller particles [44]. The relative humidity of the drying gas is proportional to the temperature, and the temperature rise leads to a moisture capacity rise [45]. Increasing the air mass flow rate, for a given temperature, the air moisture capacity is rising, thus enhancing the drying rate. Regarding the air pressure influence, for a given temperature, a pressure decrease has an opposite effect in the drying rate. A lower air pressure correlates to a lower relative humidity and higher moisture capacity, as Equation (47) implies:

(47) $$RH=\frac{y_{m}P}{P^{0}}$$

where the *y_m_* term represents the mole fraction of the moisture (water) in the air, *P* is the air total pressure and *P*^0^ is the equilibrium vapor pressure, dependent solely on temperature [46].

Conclusively, as demonstrated by Figure 7b, the validated design operational space of the SD by the input data indicated in Table 5 confirms that the in silico predictive results fit perfectly onto the associating experimental data [16], whereas the yellow narrow rectangular region (design space considered) demonstrates the area of interest with accuracy, constituting the criteria set for the responses fulfilled.

### 3.4. Solubility Enhancement Prediction and Experimental Validation

For the ternary Fenofibrate/HPMC/water system, the solubility enhancement related to the factors of temperature, particle size and stabilizer addition was calculated utilizing a combination of the PC-SAFT and the Gibbs energy change calculation method. PC-SAFT was used to describe the solid–liquid phase equilibria for the ternary system, yet this method did not include the solid’s particle size dependence. It was assumed that the solubility calculated via PC-SAFT is the one before the enhancement related to the particle size comminution and thus the Gibbs dissolution energy decrease. Equation (48) describes the dissolution constant enhancement for the two equilibriums, before and after the particle size decrease. The Fenofibrate dissolution was approximated as a first-order reaction, according to experimental time profile data [23,47]:

(48) $$\frac{1}{\nu }\frac{d{\left[Fen\right]}_{aq}}{dt}=kC_{Fen.s}$$

where [*Fen*]*_aq_* is the dissolved Fenofibrate concentration, *C_Fen_*_,*s*_ is the undissolved Fenofibrate nanoparticle concentration, *k* is the reaction’s velocity constant and *v* is the corresponding reaction coefficient. Assuming *ν =* 1 and that the solid and aqueous Fenofibrate reaction coefficients are of unit rate, then the solubility enhancement is calculated via Equation (49):

(49) $$x_{2}=\frac{\frac{K_{2}}{K_{1}}ax_{1}}{a-x_{1}+\frac{K_{2}}{K_{1}}x_{1}})$$

where *x*_2_ is the enhanced solubility, *K*_2_*/K*_1_ is the dissolution-related Gibbs energy enhancement, *a* is the initial solid API concentration before the solution reaches the equilibrium and *x*_1_ is the initial solubility. 

**Figure 8:**
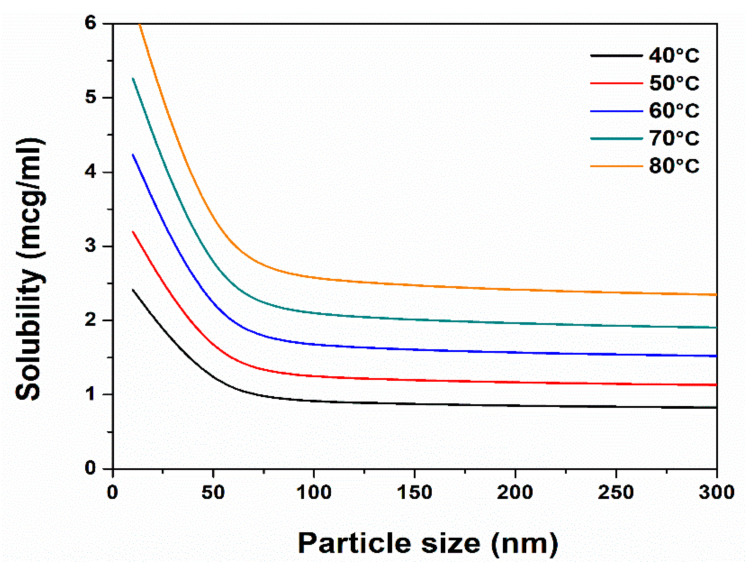
Solubility enhancement of Fenofibrate in the ternary API/HPMC/water nanosuspension.

Following the implementation of the Equations (27)–(33) and (48)–(49), the solubility enhancement related to particle reduction and temperature increase for the Fenofibrate/HPMC/water system is exhibited in Figure 8. The results show mild changes in solubility until particle sizes reach 150 nm. Temperature increase leads to significant solubility enhancement, a fact that is in accordance with the experimental data [48,49]. This solubility-to-temperature dependent relationship is attributed to the higher kinetic energy of the solvent molecules, fostering the crystal lattice bonds breakage of the solid solutes. The solubility in regard to the particle size slope changes up to 11%, ranging from 300 nm to 100 nm, but the significant change comes in particles below the 50 nm diameter threshold. This phenomenon implies that the reduced size of the produced nanocrystals offers a faster, more efficient disaggregation route towards the critical 50 nm diameter threshold.

## 4. Discussion

The material compound properties and the processes design space were initially treated separately and then integrated into the central mass and energy balance digital twin flowsheet. Fenofibrate’s crystal lattice energy was minimized to calculate the *Cij* elastic stiffness constant matrix. The latter was used as input towards the calculation of the anisotropic elastic behavior of the API, conducted by tensor and hardness statistical mechanics analysis. The Fenofibrate API was checked against anomalous elastic behavior, prohibitive of further processability. Non-negativity of the compressibility and shear behavior was unraveled. The computational results resolved the crystal lattice morphology and its related nano-elastic properties. Young’s modulus predicted the presence of bulk and crystal habit imperfections due to the high universal anisotropy index observed. The Reus Poisson’s ratio and Pugh ratio index correlated with the Gauchy pressure calculations, which quantified the ductile character of the API. The calculated Vickers hardness coefficient was plugged into the dual empirical and state equation-based estimation of the *BWI*, which was then inserted into the WMM comminution Rittinger’s law and the Rosin–Rammler distribution regime equations. Through the iterated combinatorial approach, the key process parameters of WMM were accurately predicted.

In detail, following the abovementioned pipeline, we were able to predict the critical planetary wet milling process parameters (mill diameter, material and grinding media balls mass, power and centrifugal acceleration) and the related material quality attributes (API particle size and solubility). The output results of the WMM for the related material quality attributes of the nanosuspension were then directed to the SD unit block. The Aspen Plus V9 engine converged the equations of the state and mass and energy balances of the system, providing experimentally sound predictions of the desired design space of the SD. Design space simulation prediction provided useful information for the suggested manipulation of the process parameters. In addition, the air-flow requirements for the efficient drying process were found dependent of the set inlet air temperature and pressure, as both were concluded to define the air relative humidity; thus, the relative moisture particulate capacity. The proper combination of air temperature, pressure and flow rate ascends as the triptych of critical significance for the determination not only of the API’s powder morphological characteristics but also of the practical functionality of the unit device.

In parallel, PC-SAFT modeling was enforced for the ternary nanosuspension to deliver the Helmholtz energy calculation, determining the material system’s temperature-dependent solubility prediction, by the in silico quantification of the interfacial Gibbs energy. Moreover, the phase equilibria analysis delivered by advanced PC-SAFT implementation allowed us to theoretically predict the product solubility as the critical material attribute. The solubility estimation of an API with particle sizes of only a few hundred nanometers remains a non-trivial challenge due to impracticalities related to nanosuspension inspection methods [11]. Expanding on this, there exist major difficulties not only in the separation techniques of the undissolved nanoparticles but also in the efforts to reproduce and validate experimental results. Therefore, new solubility prediction protocols performed in silico appear promising in the struggle to overcome the experimental in nature obstacles.

**Figure 9:**
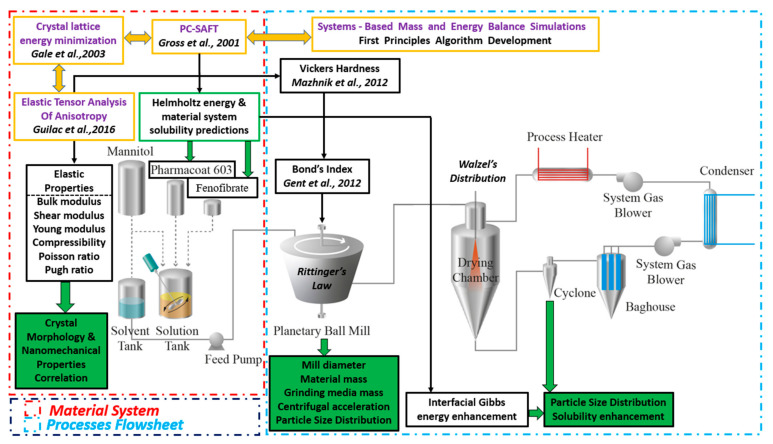
Integrating elastic tensor and PC-SAFT modeling with the systems-based Pharma 4.0 simulation to predict process operations and product specifications of ternary nanocrystalline suspensions.

All the iterated predictions regarding WMM and SD of the API nanosuspensions were validated against experimental findings from our previous and published work. The comprehensively structured proposed method and the relevant step interactions are schematically revealed in Figure 9.

Integrating PC-SAFT and statistical elastic tensor mechanics with energy and mass balance equation-solving numeric methods is a novel, data-driven industry 4.0, path combinatory method, introduced to elucidate the interactions between the key process parameters and formulation of the critical quality attributes, by first principles operability space design exploration. The significance of the proposed method is summarized by the following notations:

(a) *BWI* integration in systems-based pharmaceutics powder simulation.
(b) Equation-based numerical solution method development for predicting process design (grinding mass medium, diameter mill, centrifugal acceleration, residual moisture and drying gas temperature), and end-product material specifications (size, solubility) of WMM and SD of ternary crystalline API suspensions.
(c) Applicability of the platform for any given BCSII API ternary formulation, currently accounting for 40% of new drug formulations.
(d) Solubility prediction and dependencies solving, regarding the particle size distribution and temperature of nanocomposite material systems, crucial factors to be taken into consideration towards process development implementation and product performance assessments.
(e) Unification of the energy and mass balance processes governing equations with macroscale statistical and quantum mechanics material system data, offering exciting novel applications, especially since high-performance computing currently makes the estimation of elastic constants a viable reality.

The FDA ICH Q8-11 directives support the proposed Pharma 4.0 paradigm deployment by preaching the alignment of first principles and descriptive approaches, in order to enforce a more pragmatic manipulation of the physicochemical properties by processes both understandable and scalable on demand. Although there is a distance still to be traversed and discrepancies still to be encountered, this paradigm shift looms over the horizon, appearing more feasible than ever.

## Figures and Tables

**Table 1:** Pure component PC-SAFT parameters considered in this work.

Substance	*m_i_* (-)	*σ_i_* (Å)	*ε_i_ k*^−1^ (K)	*ε^AiBi^ k*^−1^ (K)	*κ^AiBi^* (-)	Indexed
Fenofibrate	3.85	4.76	0	0	0.02	[25]
HPMC (Pharmacoat 603)	595.4	2.88	298	1602.3	0.02	[26,27]
Water	1	3	366	2500.7	0.035	[22]

**Table 2:** PC-SAFT binary interaction parameters (*k_ij_*) considered in this work.

*i*–*j*	*k_ij_*
Fenofibrate-HPMC	0.01
Fenofibrate-Water	0
HPMC-Water	0.08

**Table 3:** Calculated mechanical properties of Fenofibrate.

Nanomechanical Stability and Anisotropy Properties											
Tool	Elastic Properties	Bulk Mod. (GPa)	Shear Mod. (GPa)	Young Mod. (GPa)	Poisson’s Ratio	Pugh Ratio	Linear Compressibility (TPa^−1^)	UAI	Hardness (GPa)	Vickers Hardness	Fracture Toughness (MPa/m^2^)
Elate	Average	9.44	4.63	11.94	0.289		0.12	4.94		0.61	0.02
ElaTools	Voigt	10.45	6.11	15.34	0.2554	1.7106	108.475	4.9368	0.901		
ElaTools	Reuss	8.43	3.15	8.404	0.363	2.6754	−4.513		0.465		
ElaTools	Average (Hill)	9.44	4.63	11.872	0.3092	2.0388			0.683		

**Table 4:** Aspen Plus V9 Fenofibrate simulation results in comparison with the experimental results [16,42] *.

Process Parameters	Aspen Plus V9	Experimental
Mill diameter	15 cm	14 cm
Rotation speed	691 rpm	450 rpm
Solution material mass	10 g	10 g
Grinding balls mass	68 g	70 g
D80 initial	100 ± 25 μm	100 μm
Centrifugal acceleration	95G	95G
Final size range	300–500 nm	300–500 nm

* https://www.fritsch-international.com/sample-preparation/milling/planetary-mills/details/product/pulverisette-7-premium-line/technical-details/, accessed on 9 September 2021.

**Table 5:** Factorial sensitivity analysis parameters used for the SD design space estimation and experimental validation.

Parameters	Run 1	Run 2	Run 3	Run 4	Run 5
F_air_, 1 (kg/h)	2500	2500	1500	2500	1500
T_air_,1 (°C)	116	116	145	116	116
Dn (mm)	0.007	0.007	0.007	0.03	0.007
P_air_,1 (bar)	6	4.5	4.5	4.5	4.5

## Data Availability

Not applicable.
